# Differential subcellular and extracellular localisations of proteins required for insulin-like growth factor- and extracellular matrix-induced signalling events in breast cancer progression

**DOI:** 10.1186/1471-2407-14-627

**Published:** 2014-08-29

**Authors:** Helen C Plant, Abhishek S Kashyap, Kerry J Manton, Brett G Hollier, Cameron P Hurst, Sandra R Stein, Glenn D Francis, Geoffrey F Beadle, Zee Upton, David I Leavesley

**Affiliations:** Tissue Repair and Regeneration Program, Institute of Health and Biomedical Innovation, Queensland University of Technology, Brisbane, Australia; Academic Clinical Research Office, Faculty of Medicine, Khon Kaen University, Khon Kaen, Thailand; Pathology Queensland, Royal Brisbane and Women’s Hospital, Brisbane, Australia; Royal Brisbane and Women’s Hospital, Brisbane, Australia

**Keywords:** Biomarker, Breast cancer, Extracellular matrix, Insulin-like growth factor, Metastasis, Vitronectin

## Abstract

**Background:**

Cancer metastasis is the main contributor to breast cancer fatalities as women with the metastatic disease have poorer survival outcomes than women with localised breast cancers. There is an urgent need to develop appropriate prognostic methods to stratify patients based on the propensities of their cancers to metastasise. The insulin-like growth factor (IGF)-I: IGF binding protein (IGFBP):vitronectin complexes have been shown to stimulate changes in gene expression favouring increased breast cancer cell survival and a migratory phenotype. We therefore investigated the prognostic potential of these IGF- and extracellular matrix (ECM) interaction-induced proteins in the early identification of breast cancers with a propensity to metastasise using patient-derived tissue microarrays.

**Methods:**

Semiquantitative immunohistochemistry analyses were performed to compare the extracellular and subcellular distribution of IGF- and ECM-induced signalling proteins among matched normal, primary cancer and metastatic cancer formalin-fixed paraffin-embedded breast tissue samples.

**Results:**

The IGF- and ECM-induced signalling proteins were differentially expressed between subcellular and extracellular localisations. Vitronectin and IGFBP-5 immunoreactivity was lower while β_1_ integrin immunoreactivity was higher in the stroma surrounding metastatic cancer tissues, as compared to normal breast and primary cancer stromal tissues. Similarly, immunoreactive stratifin was found to be increased in the stroma of primary as well as metastatic breast tissues. Immunoreactive fibronectin and β_1_ integrin was found to be highly expressed at the leading edge of tumours. Based on the immunoreactivity it was apparent that the cell signalling proteins AKT1 and ERK1/2 shuffled from the nucleus to the cytoplasm with tumour progression.

**Conclusion:**

This is the first in-depth, compartmentalised analysis of the distribution of IGF- and ECM-induced signalling proteins in metastatic breast cancers. This study has provided insights into the changing pattern of cellular localisation and expression of IGF- and ECM-induced signalling proteins in different stages of breast cancer. The differential distribution of these biomarkers could provide important prognostic and predictive indicators that may assist the clinical management of breast disease, namely in the early identification of cancers with a propensity to metastasise, and/or recur following adjuvant therapy.

**Electronic supplementary material:**

The online version of this article (doi:10.1186/1471-2407-14-627) contains supplementary material, which is available to authorized users.

## Background

Experimental and clinical evidence has implicated a role for the insulin-like growth factor (IGF) axis in cancer progression [1]. In fact a number of inhibitors of, and antibodies directed against, the IGF type I receptor (IGF-IR) have been reported to show anti-tumour activity *in vitro* and *in vivo*, and are currently in clinical trials [2]. These studies, combined with many others, have highlighted the complexity of the dysregulation of the IGF system in cancers. Simply targeting the IGF-IR or the IGF system in isolation may therefore not be the most efficacious strategy for treating this disease; more complex therapeutic approaches to target the IGF system and prevent tumorigenesis and, in particular, metastasis, are likely to be required.

Cancer metastasis is the main contributor to breast cancer fatalities [3]. Women with metastatic breast cancers have considerably poorer survival outcomes than women whose cancers are localised to the breast [4, 5]. Adjuvant systemic therapies for patients with breast cancer metastasis remain palliative [3]. Understanding the processes underpinning the progression of breast cancer, identifying patients likely to develop metastases and developing strategies to prevent the secondary spread of cancers are of significant clinical and financial relevance. There has also been a growing urgency to create cost-effective and appropriate prognostic methods that can accurately resolve those patients with a poor prognosis that require more intense treatment regimes. The prognostic methods currently available are unable to adequately address this issue [6, 7].

A critical component that is often overlooked during the identification and analysis of prognostic biomarkers, and one which may explain the inability to develop adequate prognostic techniques thus far, is the interplay between tumour cells, the surrounding microenvironment and the growth factors present in this milieu. Cellular attachment and interactions with the extracellular matrix (ECM) regulate biological responses vital for tumour progression. Considerable evidence indicates that interactions between proteins required for IGF-induced signalling events and those within the ECM contribute to processes leading to cancer progression. Studies by Kricker *et al.* [8] found that IGF-I stimulates migration of MCF-7 breast cancer cells when bound to the ECM protein vitronectin (VN) indirectly through the presence of IGF binding proteins (IGFBPs). The presence of function blocking antibodies against IGF-IR and VN-binding integrins abolished the enhanced migration of these cells [9], while, the overexpression of total-akt/protein kinase B (AKT) and phosphorylated-akt/protein kinase B (P-AKT) enhanced IGF-I: IGFBP:VN-stimulated migration [9]. Gene microarray technology has also been applied to elucidate the molecular mechanisms involved in IGF-I: IGFBP:VN-stimulated migration of breast cancer cells in *in vitro* cell based assays [10]. These studies have identified a number of genes, including Stratifin (SFN), enhancer-of-split and hairy-related protein 2 (Sharp-2), Tissue Factor, Claudin-1 (CLDN1), that are uniquely regulated by the IGF-I: IGFBP:VN complex. The genes are known for their roles in migration, invasion as well as cell survival. However, to date the effects of IGF and ECM protein interactions on the dissemination and progression of breast cancer *in vivo* are unclear. Given this, we chose to investigate the clinical relevance of proteins required for IGF-induced signalling events and those within the ECM for the development and progression of breast cancer, as well as investigate these proteins as potential prognostic biomarkers.

## Methods

### Human ethics approval

Ethical approval for this work was obtained from the Queensland University of Technology, Australia (0800000565), the Princess Alexandra Hospital Australia (2005/163), Royal Brisbane & Women’s Hospital, Australia (PR07/004) and Queensland Institute of Medical Research, Australia (P716). This project utilised archived human tissue samples collected between January 1970 and June 2005. The human tissue samples and patients records were collected as a routine part of clinical management of the breast disease. Patient consent was not required. All patient clinical information was obtained from the Queensland Cancer Registery (Australia) in a de-identified and encoded manner. Approval to use these samples and data was sought from Dr Glenn Francis and Queensland Health (Australia).

### Selection of patient specimen

This project utilised formalin-fixed paraffin-embedded (FFPE) archival breast carcinoma specimens from 91 women who presented with metastatic breast carcinoma (refer to Additional file 1 and Additional file 2 for further details). These specimens were surgically removed from the breast and axillary lymph nodes (LNs). For each patient, tissues containing normal breast epithelial ducts, primary breast carcinoma and LN metastasis were identified from haematoxylin and eosin stained sections. Cores containing DCIS tissues were omitted due to low samples numbers. Details on the selected patient cohort are provided in Additional file 3 and Additional file 4.

### Tissue microarray (TMA) construction

See details of TMA construction in Additional file 5. Where possible, the TMA cores were obtained from the leading edge of the tumour, thought to be where interactions between ligands in the ECM and the cancer cells were more likely to have functional significance [11].

### Candidate biomarkers

The candidate molecules selected for this investigation were: IGFBP-5, VN, fibronectin (FN), α_v_ integrin, β_1_ integrin, total-akt/protein kinase B 1 (Total-AKT1), P-AKT (Ser473), extracellular signal-related kinase-1 and extracellular signal-related kinase-2 (ERK1/2), phosphorylated-extracellular signal-related kinase-1 and extracellular signal-related kinase-2 (P-ERK1/2) (Thr202/Thr204), SHARP-2 and SFN. Oestrogen receptor (ER), progesterone receptor (PR) and HER2 were also selected for investigation.

### Immunohistochemistry (IHC)

The candidate markers were detected using commercially documented antibodies based upon prior independent validation for immunohistological applications in FFPE sections. Refer to Additional file 6 and Additional file 7 for specific details on the antibodies and IHC optimisation protocols, respectively.

### Distiller: a secure, web based, flexible information management system

The virtual TMA slide files created using the NanoZoomer 2.0 series (Hamamatsu^®^, Hamamatsu City, Shizuoka Pref., Japan) digital slide scanner and scanning software NDP.scan 2.0 series (Hamamatsu^®^) were uploaded into Distiller (SlidePath Ltd Digital Pathology Solutions, Santry, Dublin, Ireland) for image analysis. Distiller was used to facilitate the integration of clinical records, research data, digital TMA slides and different data types into a hierarchical database (see Additional file 8 for information).

### Scoring immunohistochemical immunoreactivity

The digital TMA images were examined and scored by trained anatomical pathology (AP) registrars without prior knowledge of the patient’s clinical data (i.e. ‘blind’) within the Distiller framework. If there were no pathologists available to score the TMAs, they were scored by Helen C Plant. Qualitative differences in the immunoreactivity of the proteins within the cytoplasm, nucleus and membrane of the cells were determined for each TMA core containing either normal breast epithelial ducts (normal), primary breast carcinoma (primary) or metastatic breast carcinoma (LN met). Qualitative differences in staining of the stromal cells and ECM adjacent to normal, primary and LN met tissue were also recorded. Protein immunoreactivity was evaluated semiquantitatively using five scoring methods. These included: presence of protein immunoreactivity; intensity of protein immunoreactivity; percentage of cells with protein immunoreactivity; percentage class and quickscore (*Q score*) scoring method [12]. Details on these scoring methods and data consolidation strategies are listed in Additional file 9.

### Statistical data analysis

PASW Statistics 18 version 18.0.2 (SPSS, IBM Corporation, Chicago, Illinois, USA) was used to evaluate statistical confidence of the data. The choice of test of association for the five scoring methods of protein immunoreactivity depended on the measurement scale of the scoring method. *Presence* is a binary outcome (present/absent) hence Pearson’s *χ*^2^ test of independence was used. For the ordinal scaled *intensity*, a Kruskal-Wallis test was employed to determine if any of the groups demonstrated differences. No protected rank-based non-parametric test exists for the post-hoc evaluation of pair-wise differences. Instead, the Mann-Whitney *U* test was used to evaluate between-groups differences with inflation of family-wise type I error being controlled using Bonferroni corrections. Finally, the remaining three measures of protein immunoreactivity, *percentage*, *percentage class* and *Q score* were all treated as quantitative outcomes and one-way Analysis of Variance (ANOVA) followed by Tukey’s HSDs for post-hoc testing was used to detect differences. As no rank-based non-parametric method exists to test for effect modification (i.e. interactions), interactions were probed by running the (one-way) Kruskal-Wallis tests for each strata of a potential effect modifier. For all tests, a significance level (*α*) of 0.05 was used, with the exception of where the Mann-Whitney U was used to test for post-hoc differences, where α_*FW*_ = α/*k* = 0.05/6 = 0.008 was used (*k* = 6 represents the number of pairwise comparisons).

## Results

The capability of the ECM and IGF system proteins to regulate cell function, and consequently tumorigenesis, is highly influenced by their spatial arrangement within and around the cell. It was observed that proteins required for IGF- and ECM-induced signalling events are differentially expressed between subcellular and extracellular localisations and that the interpretation of the protein immunoreactivity data is influenced by the scoring method applied. The results described below will only refer to the results obtained for the *Q score* scoring method [12]. The *Q score* values (*x*), including the standard deviation (*SD*) and sample numbers (*n*) for each protein across the tissue types and cellular localisation are outlined in Table 1.

**Table 1:** **Q-score values for immunoreactivity of each protein across tissue types and cellular localisation**

Protein	Cellular localisation	Type of breast tissue	***x***	***SD***	***n***
α_v_ integrin	Stroma	Normal	1.29	1.25	7
α_v_ integrin	Stroma	Primary	0.34	0.83	32
α_v_ integrin	Stroma	LN Metastasis	0.16	0.69	19
VN	Cytoplasm	Normal	0.78	1.65	23
VN	Cytoplasm	Primary	2.21	2.69	86
VN	Cytoplasm	LN Metastasis	2.44	2.38	68
VN	Stroma	Normal	7.13	3.75	23
VN	Stroma	Primary	2.84	2.73	86
VN	Stroma	LN Metastasis	0.84	2.13	68
IGFBP-5	Stroma	Normal	13.00	4.52	6
IGFBP-5	Stroma	Primary	8.49	4.18	35
IGFBP-5	Stroma	LN Metastasis	4.17	4.57	23
β_1_ integrin	Stroma	Normal	4.79	3.62	14
β_1_ integrin	Stroma	Primary	9.71	4.36	84
β_1_ integrin	Stroma	LN Metastasis	14.47	4.21	68
FN	Stroma	Normal	4.08	1.88	12
FN	Stroma	Primary	8.66	4.42	85
FN	Stroma	LN Metastasis	7.36	5.05	67
SFN	Nucleus	Normal	1.07	1.73	14
SFN	Nucleus	Primary	3.40	3.27	82
SFN	Nucleus	LN Metastasis	3.88	2.84	68
SFN	Cytoplasm	Normal	2.93	2.30	14
SFN	Cytoplasm	Primary	5.43	2.20	82
SFN	Cytoplasm	LN Metastasis	5.75	1.93	68
SFN	Stroma	Normal	0.00	0.00	14
SFN	Stroma	Primary	0.78	0.89	82
SFN	Stroma	LN Metastasis	1.12	0.95	68
SHARP-2	Nucleus	Normal	8.29	3.71	14
SHARP-2	Nucleus	Primary	5.76	5.61	86
SHARP-2	Nucleus	LN Metastasis	3.33	3.69	70
SHARP-2	Cytoplasm	Normal	6.36	4.50	14
SHARP-2	Cytoplasm	Primary	7.90	3.72	86
SHARP-2	Cytoplasm	LN Metastasis	8.80	4.57	70
T-AKT1	Nucleus	Normal	5.69	6.91	13
T-AKT1	Nucleus	Primary	3.39	4.08	85
T-AKT1	Nucleus	LN Metastasis	2.59	2.93	68
T-AKT1	Cytoplasm	Normal	7.54	3.76	13
T-AKT1	Cytoplasm	Primary	8.78	3.95	85
T-AKT1	Cytoplasm	LN Metastasis	9.37	3.67	68
P-AKT	Nucleus	Normal	13.50	6.21	16
P-AKT	Nucleus	Primary	11.51	5.52	86
P-AKT	Nucleus	LN Metastasis	9.32	5.44	68
P-AKT	Cytoplasm	Normal	1.19	1.47	16
P-AKT	Cytoplasm	Primary	2.19	2.12	86
P-AKT	Cytoplasm	LN Metastasis	2.00	2.32	68
ERK1/2	Nucleus	Normal	2.67	2.92	15
ERK1/2	Nucleus	Primary	1.77	2.76	84
ERK1/2	Nucleus	LN Metastasis	0.75	1.82	67
ERK1/2	Cytoplasm	Normal	3.73	3.31	15
ERK1/2	Cytoplasm	Primary	6.18	4.20	84
ERK1/2	Cytoplasm	LN Metastasis	5.42	3.73	67
P-ERK1/2	Nucleus	Normal	1.83	2.79	12
P-ERK1/2	Nucleus	Primary	1.89	3.47	76
P-ERK1/2	Nucleus	LN Metastasis	0.41	1.03	64
P-ERK1/2	Cytoplasm	Normal	9.67	4.46	12
P-ERK1/2	Cytoplasm	Primary	10.08	4.27	76
P-ERK1/2	Cytoplasm	LN Metastasis	9.78	3.95	64

x = Q score; SD = standard deviation; n = sample size; LN = lymph node; P = phosphorylated; T = Total.

### Changes in ECM proteins

**Figure 1:**
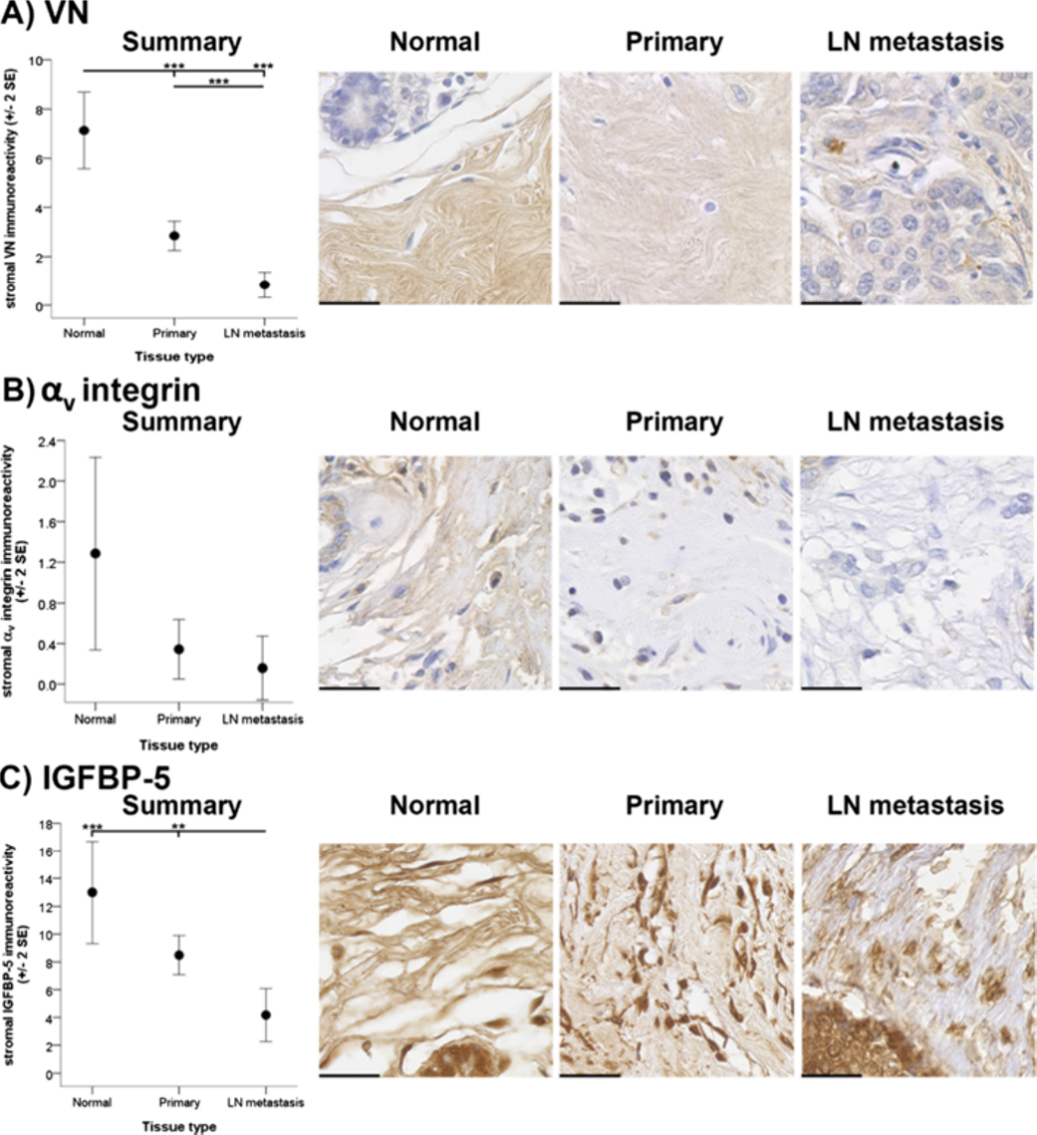
**Stromal immunoreactivity of VN, α**_**v**_ **integrin and IGFBP-5.** Immunoreactivity of VN **(A)**, α_v_ integrin **(B)** and IGFBP-5 **(C)** within the stroma surrounding normal breast (Normal), primary cancer (Primary) and LN metastasis tissues is depicted. Immunoreactivity was evaluated semiquantitatively using the *Q score* (*intensity* x *percentage class*, score: 0 – 18) method. *Intensity* of reactivity (score: 0 = negative; 1 = weak; 2 = moderate, and; 3 = strong). *Percentage class* (score: 1 = 0-4%; 2 = 5-19%; 3 = 20-39%; 4 = 40-59%; 5 = 60-79%; 6 = 80-100%). Data are displayed using the mean ± 2 standard error (*SE*). Asterisks (** and ***) indicate statistically significant differences at *p* < 0.01 and <0.001, respectively. Scale bar = 30 μm.

**Figure 2:**
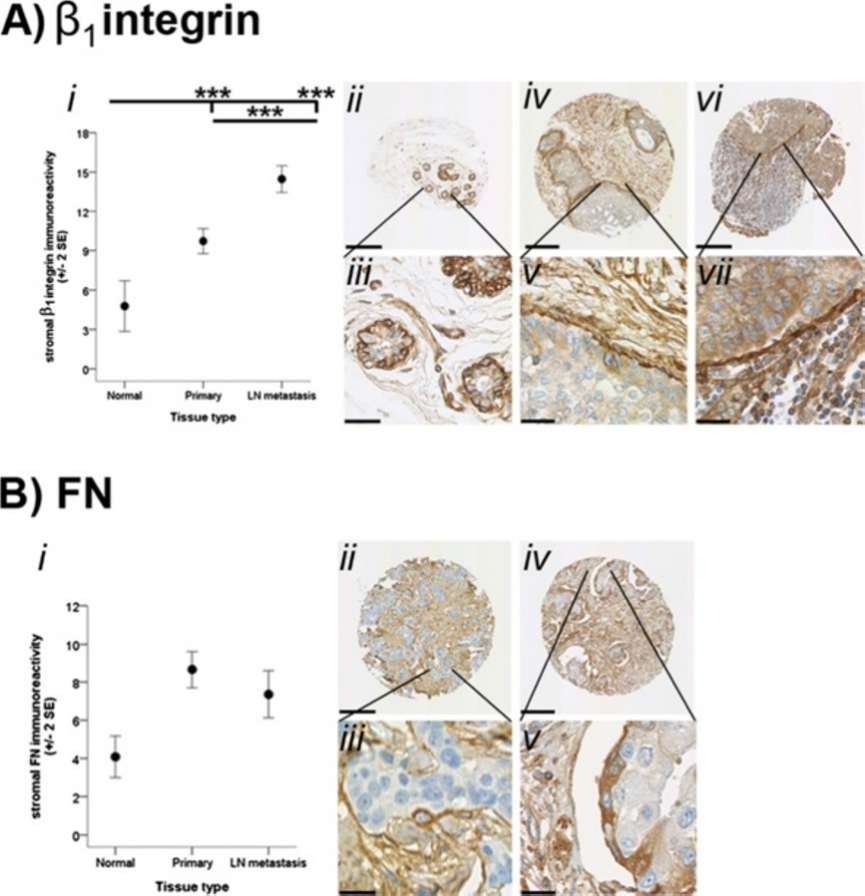
**Stromal immunoreactivity of β**_**1**_ **integrin and FN.** Immunoreactivity of β_1_ integrin is depicted in **A**. *i*) Immunoreactivity of β_1_ integrin within the stroma surrounding normal breast (Normal), primary cancer (Primary) and LN metastasis tissues. *ii* – *ix*) Representative images demonstrating distribution of β_1_ integrin in the stroma and/or cells at the leading edges of Normal (ii and iii), ductal carcinoma in situ (*iv* and *v*), Primary (vi and vii) and LN metastasis (viii and ix) tissues. Immunoreactivity of FN is depicted in **B**. *i*) Immunoreactivity of FN within the stroma surrounding Normal, Primary and LN metastasis tissues. *ii* – *v*) Representative images demonstrating distribution of β_1_ integrin immunoreactivity in the stroma and/or cells at the leading edges of Primary (*ii* and *iii*) and LN metastasis (*iv* and *v*) tissues. Immunoreactivity was evaluated semiquantitatively using the *Q score* (*intensity* x *percentage class*, score: 0 – 18) method. *Intensity* of reactivity (score: 0 = negative; 1 = weak; 2 = moderate, and; 3 = strong). *Percentage class* (score: 1 = 0-4%; 2 = 5-19%; 3 = 20-39%; 4 = 40-59%; 5 = 60-79%; 6 = 80-100%). Data are displayed using the mean ± 2 standard error (*SE*). Asterisks (* and ***) indicate statistically significant differences at *p* < 0.05 and <0.001, respectively. The scale bar for A*ii*, A*iv*, A*vi*, A*viii*, B*ii* and B*iv* is 200 μm and for A*iii*, A*v*, A*vii*, A*ix*, B*iii* and B*v* is 30 μm.

The most obvious differences in the immunoreactive distribution between normal breast, primary and metastatic cancer tissue samples was observed in proteins located in the extracellular space surrounding normal breast ducts and primary and metastatic tumours. These findings are intriguing given that the processes occurring during normal breast development are tightly regulated by the ECM and that the ability of the ECM to provide homeostatic regulation is disrupted during the development and progression of breast cancer. It was observed that the immunoreactivity of key ECM molecules, IGF regulators and integrins decreased with tumour development and/or progression. Significant differences in the immunoreactivity of stromal VN (*p* < 0.001), IGFBP-5 (*p* < 0.001) and β_1_ integrin (*p* < 0.001) within the tissue types examined were detected (Figures 1A-C and 2A*i*, respectively). Stromal IGFBP-5 and VN immunoreactivity in the metastatic cancer tissues was found to be significantly less than stromal IGFBP-5 and VN immunoreactivity in the normal breast tissues (*p* < 0.001 and *p* < 0.001, respectively) and primary cancer tissues (*p* < 0.01 and *p* < 0.001, respectively) (Figure 1C and 1A, respectively). Additionally, stromal VN immunoreactivity was greater within normal breast tissue as compared to the immunoreactivity detected in primary cancer tissues (*p* < 0.001) (Figure 1A). Despite not reaching statistical significance (*p =* 0.054), comparable trends were observed for stromal α_v_ integrin staining with increasing invasiveness of the tissue types examined (Figure 1B).

In contrast, the β_1_ integrin immunoreactivity detected in the stroma of metastatic cancer tissue was significantly higher than the β_1_ integrin immunoreactivity detected in the stroma in the normal breast (*p* < 0.001) and primary cancer (*p* < 0.001) tissue samples (Figure 2A*i*). In the primary cancer tissues, stromal reactivity of the β_1_ integrin was significantly greater than within the normal breast tissues (*p* < 0.001) (Figure 2A*i*). No statistically significant differences in FN reactivity between the various tissue types were examined (*p =* 0.094) (Figure 2B*i*). These findings suggest that VN, IGFBP-5 and α_v_ integrin reactivity in the stroma decreased while stromal β_1_ integrin immunoreactivity increased with tumour progression. Figure 2B*i* reveals trends, albeit not statistically significant, which suggest that the stromal localisation of FN differs to that of the other ECM proteins analysed.

### Tumour leading edge

Given these findings, we next investigated whether the distribution of β_1_ integrin and FN immunoreactivity within the stroma could be functionally associated with cancer invasion. FN immunoreactivity was observed throughout the stroma immediately adjacent and distal to the leading edges of each tumour (Figure 2B*ii - v*). There was also a higher presence of FN immunoreactivity both inside tumour cells at the leading edges and in the stroma directly surrounding the leading edges (Figure 2B*ii - v*). In particular, greater membrane and cytoplasmic FN was associated with tumour cells at the leading edge and in close proximity to the leading edge, in contrast to the cells within the middle of the tumour. Paralleling the distribution of FN, β_1_ integrin immunoreactivity was detected throughout the stroma immediately surrounding and distant to the leading edges of the tumours (Figure 2A*ii - ix*). There were many instances where the β_1_ integrin was also detected both inside tumour cells at the leading edges and within the stroma of the leading edges of tumours (Figure 2A*ii - ix*). Again, greater membrane β_1_ integrin immunoreactivity was observed in tumour cells at the leading edge and in close proximity to the leading edge, compared to the main bulk of the tumour. However, there were no obvious differences between the cytoplasmic expression of β_1_ integrin in cells at the leading edge of tumours and those in the centre of the tumours.

### SFN in stroma

**Figure 3:**
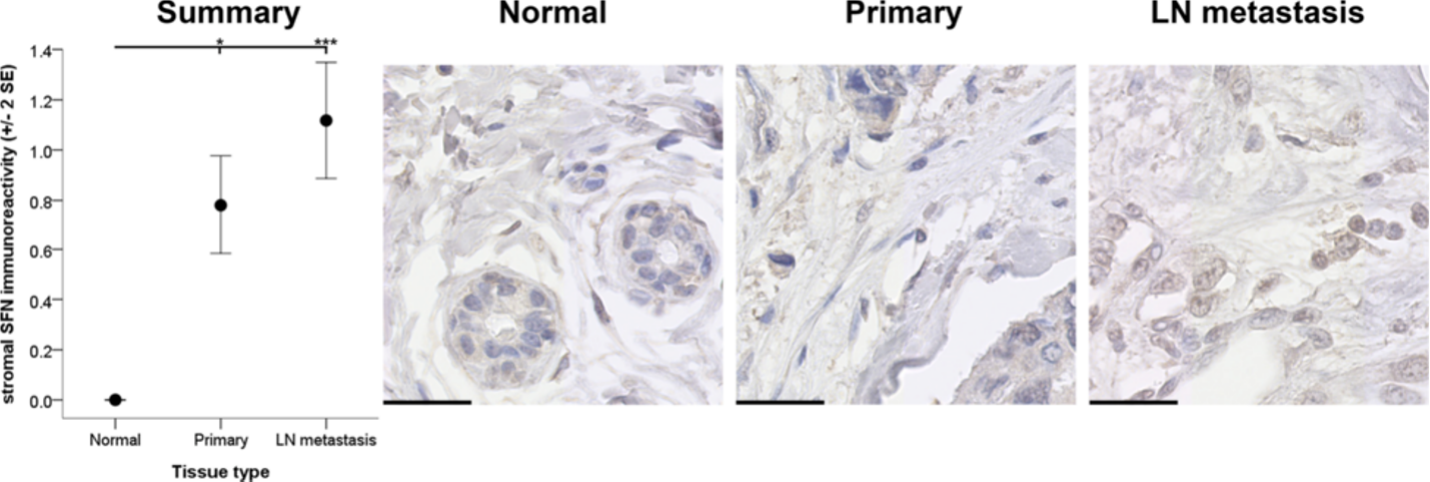
**Stromal immunoreactivity of SFN.** Immunoreactivity of SFN within the stroma surrounding normal breast (Normal), primary cancer (Primary) and LN metastasis tissues is depicted. Immunoreactivity was evaluated semiquantitatively using the *Q score* (*intensity* x *percentage class*, score: 0 – 18) method. *Intensity* of reactivity (score: 0 = negative; 1 = weak; 2 = moderate, and; 3 = strong). *Percentage class* (score: 1 = 0-4%; 2 = 5-19%; 3 = 20-39%; 4 = 40-59%; 5 = 60-79%; 6 = 80-100%). Data are displayed using the mean ± 2 standard error (*SE*). Asterisks (* and ***) indicate statistically significant differences at *p* < 0.05 and <0.001, respectively. Scale bar = 30 μm.

Significant differences were evident in the immunoreactivity of SFN in the stroma of the tissue types examined (*p* < 0.001) (Figure 3). In particular, SFN immunoreactivity scores within the stroma of normal breast tissue were significantly lower than the SFN immunoreactivity scores within stroma of primary (*p* < 0.05) and metastatic cancer tissues (*p* < 0.001). In addition, this finding suggests that SFN immunoreactivity increases in the stroma with tumour development and progression.

### Intracellular movement of cell signalling proteins

The occupation of IGF-IR and integrin molecules results in the recruitment of adapter proteins to the cell membrane and the formation of multiprotein complexes and facilitates the phosphorylation and activation of signalling cascades including AKT and MAPK [13]. The phosphorylation of AKT and MAPK impacts the cellular localisation, specificity and consequently the protein targets of these signalling molecules [14, 15]. In light of this, the immunoreactivity of Total- and Phosphorylated- AKT and ERK1/2 within the cytoplasm and nucleus of cells from normal breast, primary and metastatic cancers were investigated to determine their role in the downstream signalling events during the development and progression of breast cancer. The intracellular localisation of SHARP-2 and SFN species uniquely regulated by the IGF-I: IGFBP:VN complex [10], were also investigated.

**Figure 4:**
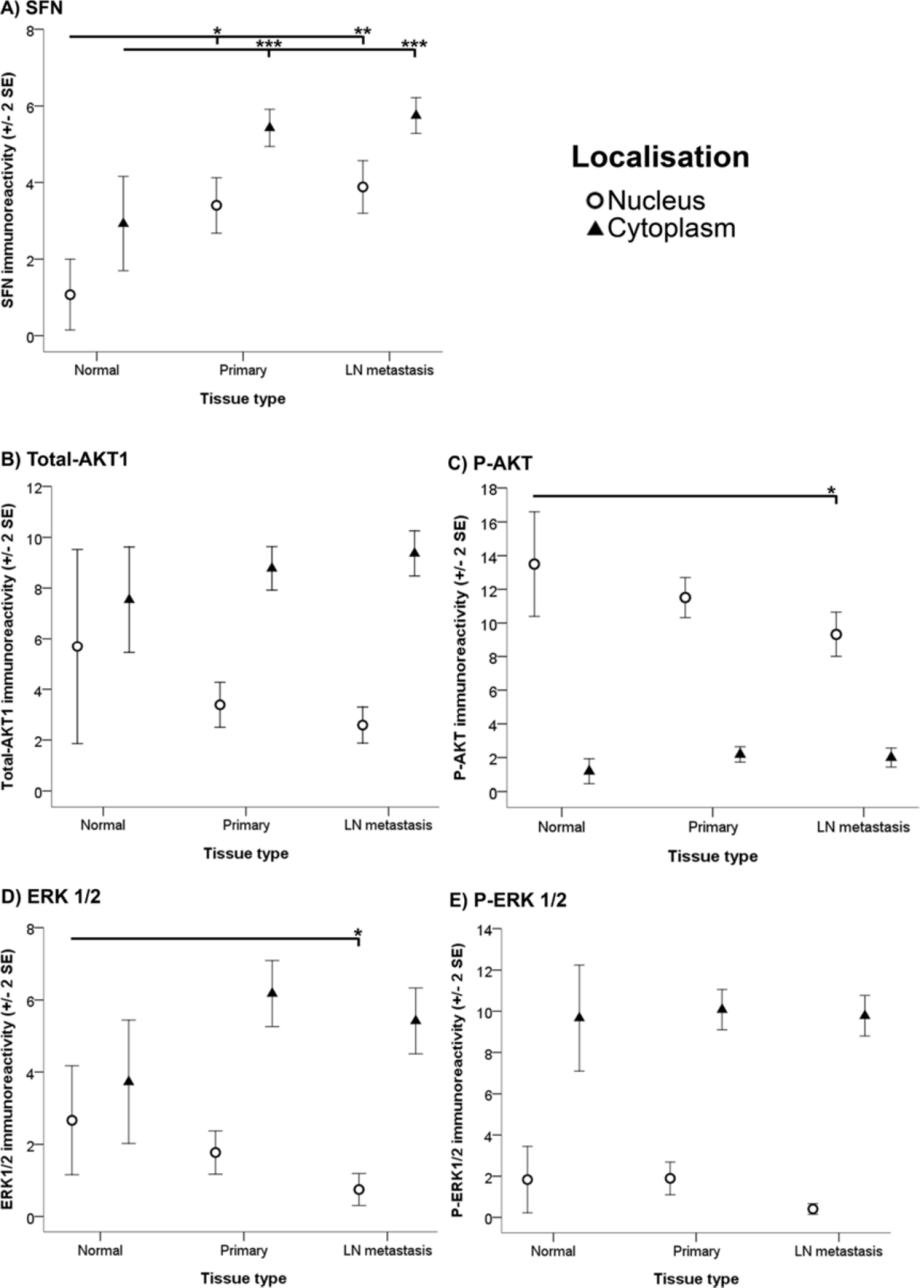
**Tissue localisation.** Differential localisation of SFN **(A)**, Total-AKT1 **(B)**, P-AKT **(C)**, ERK1/2 **(D)** and P-ERK1/2 **(E)** is depicted. Nuclear (open circles) and cytoplasmic (closed triangles) immunoreactivity within normal breast (Normal), primary cancer (Primary) and LN metastasis tissues was determined. Antibody immunoreactivity was evaluated semiquantitatively using the *Q score* (*intensity* x *percentage class*, score: 0 – 18) method. *Intensity* of reactivity (score: 0 = negative; 1 = weak; 2 = moderate, and; 3 = strong). *Percentage class* (score: 1 = 0-4%; 2 = 5-19%; 3 = 20-39%; 4 = 40-59%; 5 = 60-79%; 6 = 80-100%). Data are displayed using the mean ± 2 standard error (*SE*). Asterisks (*, ** and ***) indicate statistically significant differences at *p* < 0.05, <0.01 and <0.001, respectively.

**Figure 5:**
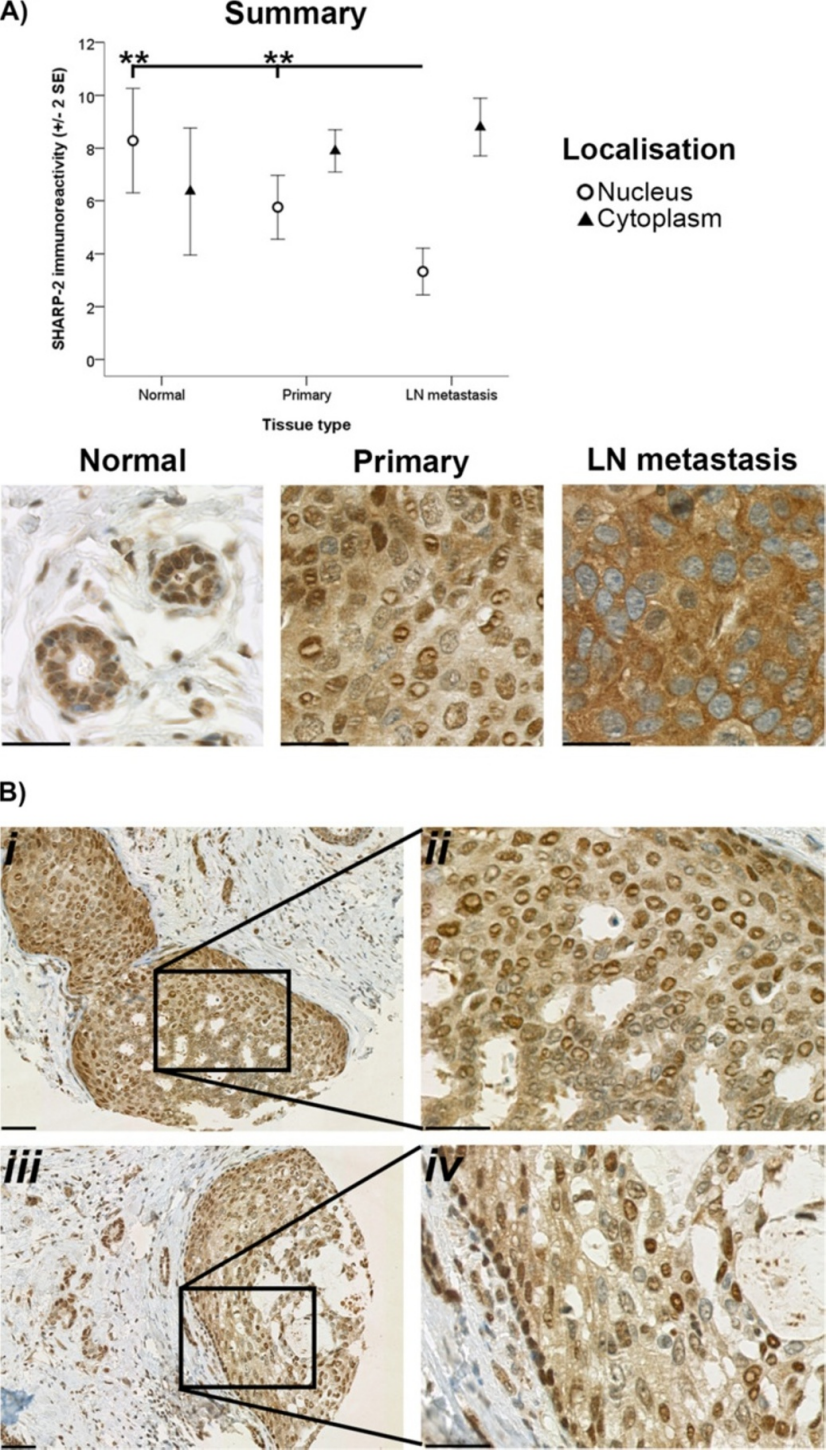
**SHARP-2 immunoreactivity.** Immunoreactivity of SHARP-2 within the nucleus (open circles) and cytoplasm (closed triangles) of cells from normal breast (Normal), primary cancer (Primary) and LN metastasis tissues is depicted in **A**. Immunoreactivity was evaluated semiquantitatively using the *Q score* (*intensity* x *percentage class*, score: 0 – 18) method. *Intensity* of reactivity (score: 0 = negative; 1 = weak; 2 = moderate, and; 3 = strong). *Percentage class* (score: 1 = 0-4%; 2 = 5-19%; 3 = 20-39%; 4 = 40-59%; 5 = 60-79%; 6 = 80-100%). Data are displayed using the mean ± 2 standard error (*SE*). Asterisks (**) indicate statistically significant differences at *p* < 0.01. Representative images demonstrating the distribution of SHARP-2 in the cancer cells of primary cancer tissue samples is depicted in **B** (*i-iv*). The scale bar for **A**, B*ii* and Bi*v* is 30 μm and for B*i* and B*iii* is 200 μm.

Significant differences in nuclear localisation of P-AKT, ERK1/2 and SHARP-2 reactivity (*p* < 0.01, *p* < 0.05 and *p* < 0.001, respectively) were detected between the normal breast, primary cancer and metastatic cancer tissues examined (Figures 4C, D and 5A). More specifically, nuclear P-AKT, ERK1/2 and SHARP-2 immunoreactivity within metastatic cancer tissues was lower than that observed within normal breast tissues (*p* < 0.05, *p* < 0.05 and *p* < 0.01, respectively) (Figures 4C, D and 5A). There was also less nuclear SHARP-2 immunoreactivity within metastatic breast tissue than within primary cancer tissues (*p* < 0.01). Thus, the nuclear localisation of P-AKT, ERK1/2 and SHARP-2 decreased with tumour development and/or progression. In contrast, Figures 4 and 5 reveal trends, albeit not statistically significant, which suggest that the cytoplasmic localisation of Total-AKT1, P-AKT and SHARP-2 may increase with tumour development and/or progression. Additionally, in many of the primary cancer and metastatic (data not shown) tissues in this study there was more nuclear immunoreactivity for SHARP-2 at the periphery of the tumour than was evident in the centre of the tumour (Figure 5B).

We further observed that differences in nuclear and cytoplasmic SFN reactivity (*p* < 0.05 and *p* < 0.001, respectively) between the normal breast, primary cancer and metastatic cancer tissues were significant (Figure 4A). In particular, nuclear and cytoplasmic SFN within normal breast tissue was lower than the amount of nuclear and cytoplasmic SFN within primary cancer (*p* < 0.05 and *p* < 0.001, respectively) and metastatic cancer tissues (*p* < 0.01 and *p* < 0.001, respectively). There were no significant differences in nuclear and cytoplasmic immunoreactivity of SFN between primary and metastatic tumours.

### Internalisation of ECM proteins

**Figure 6:**
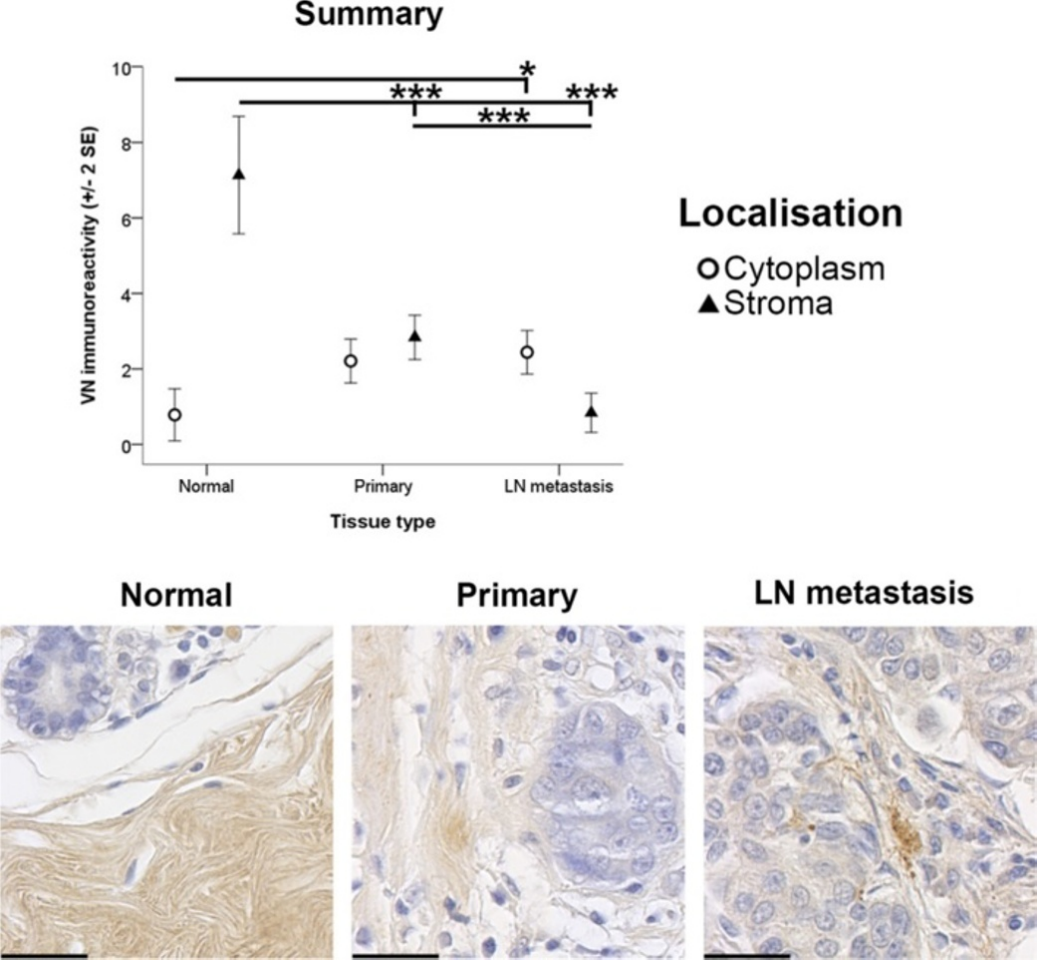
**VN immunoreactivity.** Immunoreactivity of VN within the cell cytoplasm (open circles) and the stroma (closed triangles) of normal breast (Normal), primary cancer (Primary) and LN metastasis tissues is depicted. Immunoreactivity was evaluated semiquantitatively using the *Q score* (*intensity* x *percentage class*, score: 0 – 18) method. *Intensity* of reactivity (score: 0 = negative; 1 = weak; 2 = moderate, and; 3 = strong). *Percentage class* (score: 1 = 0-4%; 2 = 5-19%; 3 = 20-39%; 4 = 40-59%; 5 = 60-79%; 6 = 80-100%). Data are displayed using the mean ± 2 standard error (*SE*). Asterisks (* and ***) indicate statistically significant differences at *p* < 0.05 and <0.001, respectively. Scale bar = 30 μm.

Our data also provides evidence that ECM molecules are internalised during breast cancer development and metastasis. Statistically significant differences in cytoplasmic (*p* < 0.05) VN immunoreactivity was observed between the specific tissue types examined in this study (Figure 6). In particular, lower cytoplasmic VN immunoreactivity was observed in normal breast tissues than cytoplasmic VN immunoreactivity within metastatic cancer tissues (*p* < 0.05) (Figure 6). As such, our data indicates that VN redistributes to the cytoplasm with tumour progression.

## Discussion

In this study a change in the immunoreactivity of key ECM molecules, IGF regulators and integrins was observed with breast tumour progression. These observations suggest that the ECM surrounding normal breast ductal structures is remodelled during tumour development and progression. The ECM protein FN and the cell surface β_1_ integrin (the FN-binding receptor) were found to be highly expressed along the leading edge of many primary tumours in the present study. Interestingly, FN is implicated in epithelial-to-mesenchymal transition (EMT) [16] and both the β_1_ integrin and FN are required in the formation of lamellipodia, filopodia and invadopodia [17, 18]; potentially supporting their role in ECM remodelling and subsequent tumour cell invasion [19]. Indeed, the β_1_ integrin has been reported to be more highly expressed in primary tumours with LN metastases [20] and with poor survival outcomes [21]. This fits in well with our findings that suggest an increase in β_1_ integrin immunoreactivity with increasing invasiveness. Various proteases, including matrix metalloproteinases (MMPs) have been implicated in ECM remodelling events that allow cancer cells to migrate [22]. It has been shown that the expression, activity and/or internalisation of MMPs is regulated by integrin-ECM interactions in endothelial cells [23]. Integrins, such as the α_v_β_3_ integrin, cooperate with MMPs to regulate breast cancer cell migration [24]. Interestingly, IGF-I:VN: IGFBP-5-stimulated breast cell migration, which requires the IGF-IR and VN-binding integrins [9], can regulate the gene expression of proteases such as MMP13, MMP7, ADAMTS5, CPM and protease inhibitors such as SERPINE1 and TRP1 ([10] and supplementary data from [10]).

The data we report here has also provided evidence that ECM molecules and their associated membrane-bound receptors, the integrins, are internalised during breast cancer development and metastasis. Step-wise increases in cytoplasmic and concomitant decreases in stromal immunoreactivity of VN and the α_v_ integrin (data not shown) were evident between normal breast, primary and metastatic cancer tissues. As described previously, VN can be internalised by integrin receptor-mediated endocytosis and degraded within the lysosomes [25]. There is also evidence indicating that the VN-binding α_v_ integrin can be recycled to the cell membrane through intracellular signals [26]. This decrease in the stromal VN and the concomitant increase in the cytoplasmic VN with breast cancer progression suggests a potential re-shuffling or trafficking of VN from the tumour stroma to the tumour cell cytoplasm with increasing invasiveness of the tumour-type.

Given the importance of the phosphoinositol 3-kinase (PI3K) and mitogen-activated protein kinase (MAPK) signalling pathways in IGF-I: IGFBP:VN-stimulated migration of breast cancer cells *in vitro* [9], and in IGF-I-stimulated ECM re-modelling [27] and EMT events [27, 28], protein intermediates within these pathways were also investigated. Decreases in the nuclear immunoreactivity and increases in the cytoplasmic immunoreactivity in breast cancer tissues were observed in this study. We propose a number of explanations for these findings: namely, the preferential activation of substrates in the cytoplasm (of cancer cells) rather than in the nucleus; enzyme-mediated dephosphorylation; and protein internalisation. Both AKT1 and MAPK contain transportation signals which potentially enable their movement throughout a cell [29, 30]. Defects in these transportation signals during cancer tumorigenesis might explain the results of this study. Ras homolog gene family member B (RhoB), which has been shown to influence the trafficking of Total- and P-AKT in primary human endothelial cells [31], may impede the import of Total- and P-AKT into or promote the export of Total- and P-AKT from the nucleus of breast cancer cells; resulting in an accumulation of AKT in cytoplasmic compartments. Phospho-kinases, such as MAPK, are not necessarily required to enter the nucleus to regulate gene transcription. In fact, the activation of transcription factors in the cytoplasm and their movement into the nucleus for transcriptional control [32] has been reported. The duration and strength of AKT and MAPK signalling in breast epithelial cells can also be regulated in different subcellular locations through the action of various cytoplasmic and nuclear phosphatases [33]. Indeed, phosphatase and tensin homolog (PTEN), a dual lipid and protein phosphatase, can be localised to the cell nucleus [34], and if functional, may therefore de-phosphorylate nuclear AKT. As discussed by Tzivion *et al.* [35], the ability of a protein to interact with modifying enzymes, such as phosphatases, can be influenced by the presence of 14-3-3 proteins.

Intriguingly, 14-3-3 proteins, including SFN, have been shown to regulate the cytoplasmic sequestration and nuclear retention of cell cycle regulators, many of which are associated with and downstream of the PI3K pathway [36]. It is highly likely that 14-3-3 proteins, such as SFN, may regulate similar sequestration events for P-AKT itself. It was intriguing to find that the increases in the nuclear and cytoplasmic immunoreactivity of SFN with breast cancer development and progression as measured in this study correlated with the increase in mRNA expression of SFN reported by Kashyap *et al.* [10]. In contrast, other studies have reported the downregulation of SFN expression in breast cancers [37, 38]. However, Neal *et al.* [39] showed that overexpression of SFN reduces the overall and disease-free survival of breast cancer patients and is able to predict which patients have a high susceptibility to develop metastasis. Interestingly, it has been proposed that 14-3-3 proteins are important regulators of external environmental signals by eliciting positive and negative effects on the IGF signalling pathway. The ability of 14-3-3 proteins to bind phospho-serine enables them to bind to the IGF-IR [40]. Yang *et al.* [41] have shown that SFN can also bind to and inhibit the activity of AKT, preventing AKT-mediated cellular events. They also indicate that SFN expression was inversely correlated with P-AKT expression [41]; this supports our findings of decreases in nuclear P-AKT and increases in intracellular SFN with tumour development and progression.

We were intrigued to find that SFN was differentially expressed within the stroma surrounding the tissue types examined. Although previous reports of SFN expression have been limited to the cytoplasm of malignant breast cells [38], *in vitro* evidence indicates that SFN can be excreted by keratinocytes into the pericellular matrix [42]. Extracellular SFN is a key regulator of MMP function and ECM degradation. Studies have shown that following the release of SFN from keratinocytes, MMP-1 mRNA [43] and MMP-1 protein synthesis [44] increases in dermal fibroblasts. Increases in mRNA encoding the β_1_ integrin have also been observed in dermal fibroblasts after treatment with SFN, or in co-cultures with keratinocytes known to release SFN [45]. Under the same conditions the expression of many ECM molecules, including collagen type I, FN and α_1_ integrin, decreases. This collective evidence suggests that it is highly likely that SFN may mediate similar functions regulating the degradation of ECM during epithelial tumour development and progression.

We also observed step-wise increases in cytoplasmic and decreases in nuclear immunoreactivity of SHARP-2 between the normal breast, primary and metastatic cancer tissues; this may be explained by nucleocytoplasmic shuttling events. SHARP-2 is known to possess a functional nuclear export sequence (NES) and two nuclear localisation signal (NLS) motifs [46]. A study by Ivanova *et al.* [46] suggests that SHARP-2 may be required in the nucleus of proliferating and differentiating cells to regulate gene transcription after stimulation by an external factor. They also propose that SHARP-2 may be sequestered in the cytoplasm following cell differentiation.

In summary, we have reported changes in the temporal and spatial distribution of IGF- and ECM-induced signalling proteins that occur during breast cancer metastasis. Specifically, our findings provide further evidence that the ECM surrounding normal breast ductal structures is remodelled during tumour development and progression, and that FN and the β_1_ integrin are important for the formation of invadopodia and for the epithelial-to-mesenchymal transition events (shown by others [47]) to support dissemination. Analysis of stromal and subcellular SFN immunoreactivity suggested a causal relationship in ECM remodelling events and the localisation and activity of proteins important for IGF- and ECM-induced signalling cascades. It also appears plausible that in cells at the leading edge of tumours, SHARP-2 moves into the nucleus to repress the transcription of genes associated with the hypoxic response.

Collectively the above data highlight the possibility that there are broader biological implications of, and explanations for, the differential immunoreactivity of IGF signalling and ECM components in the stroma and/or in subcellular locations within normal breast, primary breast cancer and metastatic breast cancers. This is highly pertinent given that protein function and protein localisation are closely correlated. Studies have also shown that accounting for protein localisation can be an essential requirement to identifying correlations with other proteins when applying the IHC technique [48]. To date, very few studies have evaluated the prognostic significance of differential protein distribution within diagnostic breast cancer tissue samples. Early studies do, however, suggest that specific locations of specific proteins associated with the IGF signalling cascade and the ECM have shown potential as markers for patient prognosis and therapeutic response [49, 50]. We argue that to date the potential of many molecular species to serve as markers of patient prognosis and therapeutic response is being missed by overlooking their subcellular/extracellular distribution. In view of this, we recommend a more complete analysis of protein localisation within diagnostic pathology and improvements to reporting and inclusion of protein localisation in routine pathological examinations as our data indicates the potential role of protein localisation in the progression of disease.

## Conclusions

There is potential that the cellular and ECM events outlined herein could be manipulated to provide clinical benefits and improve the clinical management of breast cancer. In particular, may lead to early prognostic and predictive identification of patients with poor survival outcomes. However, prior to this occurring, the prognostic significance of the cellular and ECM events reported in this study must be identified.

## Authors’ information

Helen C Plant and Abhishek S Kashyap are co-first authors.

## Electronic supplementary material

Additional file 1: **The actual number of normal breast epithelial duct, ductal carcinoma in situ (DCIS), primary breast carcinoma and/or lymph node (LN) metastasis tissues (n).** (DOCX 21 KB)

Additional file 2: **The expected number of normal breast epithelial duct, ductal carcinoma in situ (DCIS), primary breast carcinoma and/or lymph node (LN) metastasis tissues (n).** (DOCX 22 KB)

Additional file 3: **The clinico-pathological and survival data for patients.** (DOCX 20 KB)

Additional file 4: **The oestrogen receptor (ER), progesterone receptor (PR) and human epidermal growth factor receptor-2 (HER2) status data for patients.** (DOCX 20 KB)

Additional file 5: **Working with tissue microarray (TMA). Describes the steps involved in TMA construction, template editing, design computation, punch area selection and heat cycling.** (DOCX 21 KB)

Additional file 6: **Product and supplier details for the antibodies used in this study.** (DOCX 16 KB)

Additional file 7: **Immunohistochemistry (IHC) conditions for antigen detection.** (DOCX 17 KB)

Additional file 8: **Detailed information on steps involved in using the program Distiller.** (DOCX 17 KB)

Additional file 9: **Semiquantitative evaluation of immunohistochemical immunoreactivity and consolidation of TMA scoring.** (DOCX 18 KB)
