# Supplementary material for: Differential subcellular and extracellular localisations of proteins required for insulin-like growth factor- and extracellular matrix-induced signalling events in breast cancer progression
Source: BMC Cancer. 2014 Aug 29;14:627. doi: 10.1186/1471-2407-14-627 (PMC4158058; doi:10.1186/1471-2407-14-627)
Supplement: Supplementary file 1 — Additional file 1: The actual number of normal breast epithelial duct, ductal carcinoma in situ (DCIS), primary breast carcinoma and/or lymph node (LN) metastasis tissues (n). (DOCX 21 KB) [file 12885_2013_4813_MOESM1_ESM.docx]

**Manuscript title:** Differential subcellular and extracellular localisations of proteins required for insulin-like growth factor- and extracellular matrix-induced signalling events in breast cancer progression.

**Journal name:** BMC Cancer

**Additional file 1:** The actual number of normal breast epithelial duct, ductal carcinoma in situ (DCIS), primary breast carcinoma and/or lymph node (LN) metastasis tissues (n). N/A: not applicable.

| **TMA Name** | **Normal breast TMA cores (n)** | **DCIS TMA cores (n)** | **Primary cancer TMA cores (n)** | **LN metastasis TMA cores (n)** |
| --- | --- | --- | --- | --- |
| α_v_ integrin | 7 | 3 | 32 | 19 |
| β_1_ integrin | 14 | 5 | 84 | 68 |
| CLDN1 | 12 | 7 | 82 | 66 |
| ER | 16 | 2 | 73 | 64 |
| ERK1/2 | 15 | 2 | 84 | 67 |
| FN | 12 | 5 | 85 | 67 |
| HER2 | 14 | 1 | 85 | 71 |
| IGF-IR | 14 | 1 | 34 | 21 |
| IGF-IIR | 16 | 6 | 79 | 69 |
| IGFBP-5 | 6 | N/A | 35 | 23 |
| P-AKT | 16 | 2 | 86 | 68 |
| P-ERK1/2 | 12 | 5 | 76 | 64 |
| PR | 11 | 4 | 71 | 65 |
| SFN | 14 | 6 | 82 | 68 |
| SHARP-2 | 14 | 6 | 86 | 70 |
| Total-AKT1 | 13 | 4 | 85 | 68 |
| VN | 23 | 2 | 86 | 68 |

TMA = Tissue Microarray
