# Supplementary material for: Differential subcellular and extracellular localisations of proteins required for insulin-like growth factor- and extracellular matrix-induced signalling events in breast cancer progression
Source: BMC Cancer. 2014 Aug 29;14:627. doi: 10.1186/1471-2407-14-627 (PMC4158058; doi:10.1186/1471-2407-14-627)
Supplement: Supplementary file 2 — Additional file 2: The expected number of normal breast epithelial duct, ductal carcinoma in situ (DCIS), primary breast carcinoma and/or lymph node (LN) metastasis tissues (n). (DOCX 22 KB) [file 12885_2013_4813_MOESM2_ESM.docx]

**Manuscript title:** Differential subcellular and extracellular localisations of proteins required for insulin-like growth factor- and extracellular matrix-induced signalling events in breast cancer progression.

**Journal name:** BMC Cancer

**Additional file 2:** The expected number of normal breast epithelial duct, ductal carcinoma in situ (DCIS), primary breast carcinoma and/or lymph node (LN) metastasis tissues (n).

| **TMA name** | **Normal breast  TMA cores (n)** | **DCIS  TMA cores (n)** | **Primary cancer  TMA cores (n)** | **LN metastasis  TMA cores (n)** |
| --- | --- | --- | --- | --- |
| α_v_ integrin | 12 | 0 | 38 | 25 |
| β_1_ integrin | 24 | 10 | 88 | 76 |
| CLDN1 | 24 | 10 | 88 | 76 |
| ER | 24 | 10 | 88 | 76 |
| ERK1/2 | 24 | 10 | 88 | 76 |
| FN | 24 | 10 | 88 | 76 |
| HER2 | 24 | 10 | 88 | 76 |
| IGF-IR | 12 | 0 | 38 | 25 |
| IGF-IIR | 24 | 10 | 88 | 76 |
| IGFBP-5 | 12 | 0 | 38 | 25 |
| P-AKT | 24 | 10 | 88 | 76 |
| P-ERK1/2 | 24 | 10 | 88 | 76 |
| PR | 24 | 10 | 88 | 76 |
| SFN | 24 | 10 | 88 | 76 |
| SHARP-2 | 24 | 10 | 88 | 76 |
| Total-AKT1 | 24 | 10 | 88 | 76 |
| VN | 24 | 10 | 88 | 76 |

TMA = Tissue Microarray
