# Supplementary material for: Differential subcellular and extracellular localisations of proteins required for insulin-like growth factor- and extracellular matrix-induced signalling events in breast cancer progression
Source: BMC Cancer. 2014 Aug 29;14:627. doi: 10.1186/1471-2407-14-627 (PMC4158058; doi:10.1186/1471-2407-14-627)
Supplement: Supplementary file 3 — Additional file 3: The clinico-pathological and survival data for patients. (DOCX 20 KB) [file 12885_2013_4813_MOESM3_ESM.docx]

**Manuscript title:** Differential subcellular and extracellular localisations of proteins required for insulin-like growth factor- and extracellular matrix-induced signalling events in breast cancer progression.

**Journal name:** BMC Cancer

**Additional file 3:** The clinico-pathological and survival data for patients. Total number (N) and proportion (%) of patients are indicated.

|  | **Patients (N)** | **Proportion (%)** |
| --- | --- | --- |
| **Age (years)** |  |  |
| < 40 | 4 | 4 |
| 40 - <50 | 27 | 30 |
| 50 - <60 | 25 | 27 |
| 60 - <70 | 22 | 24 |
| 70 - <80 | 11 | 12 |
| 80 - <90 | 1 | 1 |
| Missing | 1 | 1 |
| Mean (± standard deviation) | 55.91 (± 10.92) |  |
| **Tumour type (No/Yes/Missing)** |  |  |
| Ductal | 6/79/6 | 7/87/7 |
| Lobular | 74/11/6 | 81/12/7 |
| Tubular | 83/2/6 | 91/2/7 |
| Pleomorphic | 83/2/6 | 91/2/7 |
| Trabecular | 84/1/6 | 92/1/7 |
| Atypical medullary | 58/1/32 | 64/1/35 |
| **Overall tumour grade** |  |  |
| 1 | 3 | 3 |
| 2 | 15 | 16 |
| 3 | 59 | 65 |
| Missing | 14 | 15 |
| **Tumour size (mm) (maximum)** |  |  |
| ≤ 10 | 4 | 4 |
| 11-20 | 21 | 23 |
| 21-30 | 26 | 29 |
| 31-40 | 15 | 16 |
| 41-50 | 7 | 8 |
| 51-60 | 3 | 3 |
| 61-70 | 3 | 3 |
| 71-80 | 1 | 1 |
| 81-90 | 1 | 1 |
| 91-100 | 1 | 1 |
| Missing | 25 | 27 |
| **Positive lymph nodes** |  |  |
| 1-3 | 42 | 46 |
| 4-9 | 20 | 22 |
| ≥10 | 27 | 30 |
| Missing | 2 | 2 |
| **Lymph node ratio** |  |  |
| ≤ 0.25 | 46 | 51 |
| >0.25, ≤ 0.75 | 32 | 35 |
| ≥ 0.75 | 10 | 11 |
| Missing | 3 | 3 |
| **Survival status** |  |  |
| Deceased (due to disease) | 44 | 48 |
| Censored | 0 | 0 |
| Deceased (due to other causes) | 1 | 1 |
| Lost at date of death follow-up | 36 | 40 |
| Lost prior to follow-up | 10 | 11 |
