# Supplementary material for: Differential subcellular and extracellular localisations of proteins required for insulin-like growth factor- and extracellular matrix-induced signalling events in breast cancer progression
Source: BMC Cancer. 2014 Aug 29;14:627. doi: 10.1186/1471-2407-14-627 (PMC4158058; doi:10.1186/1471-2407-14-627)
Supplement: Supplementary file 4 — Additional file 4: The oestrogen receptor (ER), progesterone receptor (PR) and human epidermal growth factor receptor-2 (HER2) status data for patients. (DOCX 20 KB) [file 12885_2013_4813_MOESM4_ESM.docx]

**Manuscript title:** Differential subcellular and extracellular localisations of proteins required for insulin-like growth factor- and extracellular matrix-induced signalling events in breast cancer progression.

**Journal name:** BMC Cancer

**Additional file 4:** The oestrogen receptor (ER), progesterone receptor (PR) and human epidermal growth factor receptor-2 (HER2) status data for patients. Total number (N) and proportion (%) of patients are indicated.

|  | **Patients (N)** | **Proportion (%)** |
| --- | --- | --- |
| **ER percentage** |  |  |
| 0 | 21 | 23 |
| 1 - 10 | 3 | 3 |
| 11 -20 | 3 | 3 |
| 21 - 30 | 1 | 1 |
| 31 - 40 | 0 | 0 |
| 41 - 50 | 1 | 1 |
| 51 - 60 | 3 | 3 |
| 61 - 70 | 1 | 1 |
| 71 - 80 | 6 | 7 |
| 81 - 90 | 11 | 12 |
| 91 - 100 | 23 | 25 |
| Missing | 18 | 20 |
| **ER intensity** |  |  |
| 0 | 21 | 23 |
| 1 | 12 | 13 |
| 2 | 19 | 21 |
| 3 | 21 | 23 |
| Missing | 18 | 20 |
| **PR percentage** |  |  |
| 0 | 29 | 32 |
| 1 - 10 | 10 | 11 |
| 11 -20 | 4 | 4 |
| 21 - 30 | 4 | 4 |
| 31 - 40 | 1 | 1 |
| 41 - 50 | 6 | 7 |
| 51 - 60 | 2 | 2 |
| 61 - 70 | 5 | 5 |
| 71 - 80 | 3 | 3 |
| 81 - 90 | 2 | 2 |
| 91 - 100 | 5 | 5 |
| Missing | 20 | 22 |
| **PR intensity** |  |  |
| 0 | 29 | 32 |
| 1 | 14 | 15 |
| 2 | 18 | 20 |
| 3 | 10 | 11 |
| Missing | 20 | 22 |
| **HER2 percentage** |  |  |
| 0 | 46 | 51 |
| 1 - 10 | 7 | 8 |
| 11 -20 | 4 | 4 |
| 21 - 30 | 4 | 4 |
| 31 - 40 | 1 | 1 |
| 41 - 50 | 1 | 1 |
| 51 - 60 | 2 | 2 |
| 61 - 70 | 2 | 2 |
| 71 - 80 | 4 | 4 |
| 81 - 90 | 6 | 7 |
| 91 - 100 | 7 | 8 |
| Missing | 7 | 8 |
| **HER2 intensity** |  |  |
| 0 | 46 | 51 |
| 1 | 16 | 18 |
| 2 | 7 | 8 |
| 3 | 15 | 16 |
| Missing | 7 | 7 |
