# Supplementary material for: Differential subcellular and extracellular localisations of proteins required for insulin-like growth factor- and extracellular matrix-induced signalling events in breast cancer progression
Source: BMC Cancer. 2014 Aug 29;14:627. doi: 10.1186/1471-2407-14-627 (PMC4158058; doi:10.1186/1471-2407-14-627)
Supplement: Supplementary file 5 — Additional file 5: Working with tissue microarray (TMA). Describes the steps involved in TMA construction, template editing, design computation, punch area selection and heat cycling. (DOCX 21 KB) [file 12885_2013_4813_MOESM5_ESM.docx]

**Manuscript title:** Differential subcellular and extracellular localisations of proteins required for insulin-like growth factor- and extracellular matrix-induced signalling events in breast cancer progression.

**Journal name:** BMC Cancer

**Additional file 5:** Working with tissue microarray (TMA)

***Tissue microarray (TMA) construction:*** The Galileo TMA (CK3000) Tissue Microarrayer (Integrated Systems Engineering S.r.l., Milan, Italy) using the IseTMA software program (©BioRep, Milan, Italy) and the Beecher (ATA-27) Automated Tissue Arrayer (Beecher Instruments, Inc., Sun Prairie, Wisconsin, USA) were used to construct the TMAs. The methodologies listed below are those performed whilst using the Galileo TMA Tissue Microarrayer with the IseTMA software program. TMAs constructed by the MaCHR Laboratory using the Beecher (ATA-27) Automated Tissue Arrayer were utilised for this study following the manufacturer’s instructions.

***Tissue microarray (TMA) template editing:*** TMA templates were created following the TMA grid design phase. The dimensions of the blank, recipient FFPE blocks were determined and recorded in the IseTMA software program. The dimensions of the resulting TMAs were dependent on the physical dimensions of the recipient FFPE blocks. The geometry of the array (which included the required number of rows and columns) was defined using the following criteria: the block dimensions; X and Y margins; the needle diameter (0.6 mm); the number of spots; and the spacing (or the X and Y pitch) between the spots. For this study, 0.6 mm diameter cores were selected. Rows and columns of spacing gaps were included to further aid in the orientation of the TMA. Finally, the ES 111 Optiscan^™^ Upright Microscope Stage (Prior Scientific Inc., Rockland, Massachusetts, USA) was calibrated to ensure the precise positioning of the TMA within the centre of the recipient FFPE blocks. The template file created was saved as a .csv file.

***Tissue microarray (TMA) design computation:*** Within the IseTMA software program, the TMA template file was opened and an appropriate TMA construction sequence selected. The FFPE blocks included in the study (commonly known as donor FFPE blocks) were associated with one spot or multiple spots within the TMA. To achieve this, the patient AP codes were ‘linked’ to the individual TMA cores within the TMA template. Colour codes were used to identify TMA cores associated with normal breast epithelial ducts, DCIS, primary breast carcinoma and LN metastasis. The number of replicas of the created TMA was defined. To create duplicate TMAs, two recipient FFPE block copies were selected and codes created for each copy. Each of the operations listed above were performed remotely from the arrayer. The design file created was saved as a .csv file.

***Tissue microarray (TMA) construction:*** Initially, the arrayer was calibrated and the TMA template and design files were opened. The TMA needle was calibrated to ensure the precise positioning of the paraffin blocks under the needles in the operation conditions. A high resolution ProgRes^®^ C10plus camera (JENOPTIK Optical Systems GmbH, Jena, Germany) was utilised for TMA needle calibration. The two recipient FFPE blocks were inserted into the Upright stage. The IseTMA software program provided an operating flowchart to assist in TMA construction.

***Tissue microarray (TMA) core punch area selection - Donor block and haematoxylin and eosin (H&E) slide alignment:*** Where possible, the cores were obtained from the leading edge of the tumour, thought to be where interactions between ligands in the ECM and the cancer cells were more likely to occur (Giovannucci 1999; Gladson *et al.* 1991; Tomasini-Johansson *et al.* 1994). This was achieved by inserting the donor FFPE blocks into the upright stage and choosing the donor TMA spot positions. To localise the sample, an H&E-stained tissue section was placed on top of its corresponding FFPE block. Following this, the pre-marked H&E slide was manually aligned with the donor block with reference to the monitor. This allowed for the accurate identification of the tumour edge. Once aligned, a number of donor spot positions, along the tumour’s edge (if applicable), were selected using a joystick. All of the punch positions for the donor FFPE block were saved at the same time. Subsequently, holes were prepared in the recipient FFPE blocks and sample cores were transferred from the donor FFPE block and inserted into the corresponding holes in the recipient FFPE blocks. This process was performed manually and repeated for each of the donor FFPE blocks.

***Tissue microarray (TMA) heat cycling:*** To stabilise and prevent the loss of TMA cores during sectioning and immunohistochemistry (IHC) procedures a thin layer of wax was applied to the surface of all of the created TMAs containing the sample cores from the donor FFPE blocks. This was followed by a heat cycling step which involved heating the TMAs in a conventional laboratory oven at 30 degrees Celsius (°C) for short increments of time over 24 hours.
