# Supplementary material for: Differential subcellular and extracellular localisations of proteins required for insulin-like growth factor- and extracellular matrix-induced signalling events in breast cancer progression
Source: BMC Cancer. 2014 Aug 29;14:627. doi: 10.1186/1471-2407-14-627 (PMC4158058; doi:10.1186/1471-2407-14-627)
Supplement: Supplementary file 6 — Additional file 6: Product and supplier details for the antibodies used in this study. (DOCX 16 KB) [file 12885_2013_4813_MOESM6_ESM.docx]

**Manuscript title:** Differential subcellular and extracellular localisations of proteins required for insulin-like growth factor- and extracellular matrix-induced signalling events in breast cancer progression.

**Journal name:** BMC Cancer

**Additional file 6:** Product and supplier details for the antibodies used in this study. IgG: immunoglobulin G; mAb: monoclonal antibody; pAb: polyclonal antibody, and; N/A: not applicable.

| **Antibody target** | **Supplier** | **Supplier details** | **Product Number** | **Isotype** | **Source** | **Clone** |
| --- | --- | --- | --- | --- | --- | --- |
| IGF-IRβ | Santa Cruz Biotechnology^®^ | California, USA | sc-713 | IgG | Rabbit pAb | C-20 |
| IGF-IIR | Santa Cruz Biotechnology^®^ | California, USA | sc-25462 | IgG | Rabbit pAb | H-300 |
| IGFBP-5 | R&D Systems^®^ | NSW, Australia | MAB875 | IgG_2b_ | Mouse mAb | 164503 |
| VN | Epitomics^®^ | California, USA | 1730-1 | IgG | Rabbit mAb | EP781Y |
| FN | Novocastra | NSW, Australia | NCL-FIB | IgG_1_ | Mouse mAb | 568 |
| α_v_ integrin | Calbiochem^®^ | New Jersey, USA | 407286 | IgG_1_ | Mouse mAb | 272-17E6 |
| β_1_ integrin | Abcam^®^ | Massachusetts, USA | ab3167 | IgG_1_ | Mouse mAb | 4B7R |
| Total-AKT1 | Cell Signalling Technology^®^ | Massachusetts, USA | 2967 | IgG_1_ | Mouse mAb | 2H10 |
| P-AKT (Ser473) | Cell Signalling Technology^®^ | Massachusetts, USA | 4051L | IgG_2b_ | Mouse mAb | 587F11 |
| ERK1/2 | Cell Signalling Technology^®^ | Massachusetts, USA | 4695 | IgG | Rabbit pAb | 137F5 |
| P-ERK1/2 (Thr202/Thr204) | Cell Signalling Technology^®^ | Massachusetts, USA | 9106 | IgG_1_ | Mouse mAb | E10 |
| CLDN-1 | Invitrogen™ | VIC, Australia | 18-7362 | IgG | Rabbit pAb | N/A |
| SFN | Abcam^®^ | Massachusetts, USA | 110-02810 | IgG_1_ | Mouse mAb | 3C3 |
| SHARP-2 | Sigma-Aldrich^®^ | NSW, Australia | S8443 | IgG | Rabbit pAb | N/A |
| ER | Ventana Medical Systems^®^ | Arizona, USA | 790-4325 | IgG | Rabbit mAb | SP1 |
| PR | Ventana Medical Systems^®^ | Arizona, USA | 790-4296 | IgG | Rabbit mAb | 1E2 |
| HER2 | Ventana Medical Systems^®^ | Arizona, USA | 790-2991 | IgG | Rabbit mAb | 4B5 |
