# Supplementary material for: Differential subcellular and extracellular localisations of proteins required for insulin-like growth factor- and extracellular matrix-induced signalling events in breast cancer progression
Source: BMC Cancer. 2014 Aug 29;14:627. doi: 10.1186/1471-2407-14-627 (PMC4158058; doi:10.1186/1471-2407-14-627)
Supplement: Supplementary file 7 — Additional file 7: Immunohistochemistry (IHC) conditions for antigen detection. (DOCX 17 KB) [file 12885_2013_4813_MOESM7_ESM.docx]

**Manuscript title:** Differential subcellular and extracellular localisations of proteins required for insulin-like growth factor- and extracellular matrix-induced signalling events in breast cancer progression.

**Journal name:** BMC Cancer

**Additional file 7:** Immunohistochemistry (IHC) conditions for antigen detection. Incubation times are provided in minutes (mins). °C: degrees Celsius.

| Antigen | Clone | Blocking non-specific background | | Antigen Retreival | | Antigen detection | | | | Antigen probe | HRP-Polymer | Peroxidase Chromogen conditions |
| --- | --- | --- | --- | --- | --- | --- | --- | --- | --- | --- | --- | --- |
|  |  | Endogenous peroxidase | Protein | Solution | Conditions | System | Incubation time | Dilution | Diluent |  |  |  |
| α_v_ integrin | 272-17E6 | 5 min | 10 min | Diva | 40 min at 99°C with 30 min cooling | MACH 4™ | 30 min | 1:50 | Da Vinci | 10 min | 10 min | 1 min |
| β_1_ integrin | 4B7R | Performed onboard | | Protease 1 | 10 min | ultraView™ | 32 min | 1:2500 | Da Vinci | Performed onboard | | |
| CLDN-1 | N/A | Performed onboard | | Borg | 4 min at 125°C with 30 min cooling | ultraView™ | 32 min | 1:100 | Da Vinci | Performed onboard | | |
| ER | SP1 | Performed onboard | | | | iView™ | 30 min | Pre-diluted antibody | | Performed onboard | | |
| ERK1/2 | 137F5 | 5 min | 10 min | Not Performed | | MACH 3™ | 60 min | 1:150 | Da Vinci | 15 min | 15 min | 5 min |
| FN | 568 | Performed onboard | | Reveal | 4 min at 125°C with 30 min cooling | ultraView™ | 32 min | 1:200 | Da Vinci | Performed onboard | | |
| HER2 | 4B5 | Performed onboard | | | | iView™ | 32 min | Pre-diluted antibody | | Performed onboard | | |
| IGF-IRβ | C-20 | 5 min | 10 min | Diva | 4 min at 125°C with 30 min cooling | MACH 4™ | 60 min | 1:50 | Da Vinci | 15 min | 15 min | 1 min |
| IGF-IIR | H-300 | Performed onboard | | Borg | 4 min at 125°C with 30 min cooling | ultraView™ | 32 min | 1:50 | Da Vinci | Performed onboard | | |
| IGFBP-5 | 164503 | 5 min | 10 min | Not Performed | | MACH 4™ | 30 min | 1:75 | Da Vinci | 10 min | 10 min | 1 min |
| P-AKT | 587F11 | Performed onboard | | Reveal | 4 min at 125°C with 30 min cooling | ultraView™ | 32 min | 1:50 | Da Vinci | Performed onboard | | |
| P-ERK1/2 | E10 | 5 min | 10 min | Not Performed | | MACH 4™ | 60 min | 1:100 | Da Vinci | 10 min | 15 min | 2 min |
| PR | 1E2 | Performed onboard | | | | iView™ | 30 min | Pre-diluted antibody | | Performed onboard | | |
| SFN | 3C3 | Not Performed | Not Performed | Reveal | 4 min at 125°C with 30 min cooling | MACH 4™ | 60 min | 1:50 | Da Vinci | 10 min | 15 min | 5 min |
| SHARP-2 | N/A | Performed onboard | | Reveal | 4 min at 125°C with 30 min cooling | ultraView™ | 32 min | 1:1600 | Da Vinci | Performed onboard | | |
| Total-AKT1 | 2H10 | Performed onboard | | Reveal | 4 min at 125°C with 30 min cooling | ultraView™ | 32 min | 1:50 | Da Vinci | Performed onboard | | |
| VN | EP781Y | 5 min | 10 min | Not Performed | | MACH 3™ | 30 min | 1:50 | Da Vinci | 10 min | 10 min | 4 min |
