# Supplementary material for: Differential subcellular and extracellular localisations of proteins required for insulin-like growth factor- and extracellular matrix-induced signalling events in breast cancer progression
Source: BMC Cancer. 2014 Aug 29;14:627. doi: 10.1186/1471-2407-14-627 (PMC4158058; doi:10.1186/1471-2407-14-627)
Supplement: Supplementary file 8 — Additional file 8: Detailed information on steps involved in using the program Distiller. (DOCX 17 KB) [file 12885_2013_4813_MOESM8_ESM.docx]

**Manuscript title:** Differential subcellular and extracellular localisations of proteins required for insulin-like growth factor- and extracellular matrix-induced signalling events in breast cancer progression.

**Journal name:** BMC Cancer

**Additional file 8:** Detailed information on steps involved in using the program Distiller. Authorised consent was obtained from G.D.F. to gain access to the Brisbane Database of all PAH breast cancer patients.

**Data uploading and browsing**

The ‘Browse’ tab was used to upload ‘general’ patient data files (including patient, biopsy, survival and treatment data) into Distiller. The ‘Browse’ tab was also used to find all of the demographic, biopsy, treatment, follow-up, TMA and full face biomarker information, as well as the images and scoring information for all TMA cores for each patient.

***Uploading general patient data files:*** Click on the ‘Browse’ tab > Click on the ‘Files’ tab > Upload required files.

***Browsing patient information:*** Click on the ‘Browse’ tab > Click on the ‘Data’ tab > Upload required files.

**Management of high throughput design and analysis of TMA data**

The ‘OpTMA’ and ‘Administration’ tabs were used to manage high throughput design and analysis of TMA data within the Distiller framework. This included uploading, creating, editing, approving and/or managing the TMA spot groups, TMA scoring forms, TMA maps and TMA slides.

***Creating TMA spot group:*** Click on the “Administration” Tab > Click on the “DB Admin Tab” Tab > Click on the “Create new data group” icon > Type “TMA” into the “Group type name” section > Select “TMA” in the “Parent” tab > Select “TMA Spots” in the “Table type” tab > Click on the “Submit” icon.

***Editing TMA spot group attributes:*** The mandatory TMA spot group default attributes include: Spot ID; Treatment name; Slide ID; Row Number; Column Number and Block reference. Click on the “Administration” Tab > Click on the “DB Admin Tab” Tab > Click on the TMA spot group of interest > Select “Choice” in the “Type” tab > Add treatment/stains/biomarker types.

***Creating TMA scoring form group:*** Click on the “Administration” Tab > Click on the “DB Admin Tab” Tab > Click on the “Create new data group” icon > Type “TMA Score *Antibody name*” into the “Group type name” section > Select “TMA” in the “Parent” tab > Select “TMA scoring form” in the “Table type” tab > Click on the “Submit” icon.

***Editing TMA scoring form attributes:*** *Editing the name of a TMA scoring group:* Click on the “Administration” Tab > Click on the “DB Admin Tab” Tab > Scroll down to the TMA scoring group of interest > Click on the 2^nd^ column icon “Edit name” > Click on the 2^nd^ icon “Save” button to save changes. *Modifying and adding questions for a TMA scoring group:* Click on the “Administration” Tab > Click on the “DB Admin Tab” Tab > Scroll down to the TMA scoring group of interest > Click on the 1^st^ column icon “view attributes/questions’ > Click on the “create new question” icon > Type in the question name > Select if question is unique > Select whether the question is mandatory > Select an appropriate data type > Click on the “Submit” icon. *Creating choice attributes:* Click on the “Administration” Tab > Click on the “DB Admin Tab” Tab > Scroll down to the TMA scoring group of interest > Click on the 1^st^ column icon “view attributes/questions’ > Click on the “Choice” button for the attribute of interest > Select the type of “Choice frequency” > Click on “Create new choice type” > Type in the Choice name > Type in the Choice value > Click on the “Edit/Save” icon > Choose the position of the choices.

***Managing TMA maps:*** *Uploading TMA maps:* Click on the “OpTMA” Tab > Click on the “TMA Maps” Tab > Click on the “Upload” Tab to upload a TMA map from a Microsoft Excel™ spreadsheet. *Editing and approving TMA maps*: Click on the “OpTMA” Tab > Click on the “TMA Maps” Tab > Click on the TMA map of interest > Modify/Approve where necessary.

***Managing TMA slides:*** *Uploading TMA slides for dearraying:* Click on the “OpTMA” Tab > Click on the “TMA slides” Tab > Click on tabs for the folders of interest which are located on the QUT server > Click on the link for the slide of interest > Select the TMA of interest in the “block reference” section > Click “TMA” for the “Group type name” section > Select the antibody of interest in the “treatment” section (add a new treatment name) > Select “x40” in the “Magnification” section > Select the relevant orientation (N.B. There were 8 possible scanning orientations for the slides) > Click on the “Submit” icon for dearraying to occur. *Approving TMA slides:* Click on the “OpTMA” Tab > Click on the “TMA slides” Tab > Expand the “Block Reference” tab to view the slides associated with this block reference > Click on the link under “Dearray comments” to view the predicted grid > Edit/Fix the predicted grid structures. *Editing the predicted grid structures:* The annotations (red circles) mark each core. Moving the cursor over each of the annotations will display the row and column number of each core. To reposition, redraw, delete the annotation, and/or draw new cores, right-click on the annotation. The row & column numbers are assigned manually or automatically, by regridding the slide. Regridding attempts to reconstruct the TMA grid using all the annotations including any newly created cores. To save the edited grid structure press “F5”. Recheck the dearray comments and modify if necessary. If the predicted grid matches the map, the slide can be approved. Once the grid structure has been approved, an appropriate scoring form and list of scorers are selected.

***Scoring the IHC stained TMAs:*** *Scoring TMAs:* Click on the “OpTMA” Tab > Click on the “score TMAs” Tab > Click on the appropriate block reference tab > Click on the “Score” tab to score the TMA slide > Fill in the blank spaces or highlight the appropriate choice in the TMA scoring sheet that appears of the screen > Click “Next” to proceed to the next TMA core (if necessary, click on “Previous” to go back to the previous TMA core) > Click the “F5” button to save the scoring data. *Reviewing and Re-scoring TMAs:* Click on the “OpTMA” Tab > Click on the “score TMAs” Tab > Click on the appropriate block reference tab > Click on the “Review” tab to review the scored TMA slide or to re-score the TMA slide > Review or re-score the TMA cores by filling in the spaces provided or highlighting the appropriate choice in the TMA scoring sheet that appears of the screen > Click “Next” to proceed to the next TMA core (if necessary, click on “Previous” to go back to the previous TMA core) > Click the “F5” button to save changes.

**Creating searches to extract patient, biopsy, survival, treatment and TMA scoring data**

The ‘Search’ tab was used to create and re-run old searches to extract the patient, biopsy, survival, treatment and TMA scoring data of interest from Distiller. Only the attributes or data of interest were selected to be displayed in the search results to limit the extent of data extracted for analysis.

***Creating searches:*** Click on the “Search” Tab > Click on the “Constraints” Tab > Scroll down to the Data groups section > Click on the “Patient Information” Tab > Type “P” into the “Patient ID” section > Click on the “TMA score ….” Tab for the protein of interest > Tick the appropriate protein in “Biomarker” section > Click on the “TMA” Tab > Scroll down to the “Block Reference” section > Type in the appropriate TMA design name of interest > Click on the “Output” tab > Scroll down to the Data groups section > Click on the “Patient Information” tab > Select “Select all” to highlight all fields in this data group > Click on the “Biopsy Information” tab > Select “Select all” to highlight all fields in this data group > Click on the “Pathology” tab > Select “Select all” to highlight all fields in this data group > Click on the “Treatment Information” tab > Select “Select all” to highlight all fields in this data group > Click on the “Follow up Information” tab > Select “Select all” to highlight all fields in this data group > Click on the “TMA” tab > Select “Select all” to highlight all fields in this data group > Click on the “TMA score ….” tab for the protein of interest > Select “Select all” to highlight all fields in this data group > Click on the “Results” Tab to view the search results > Click “Download” to download the search results in a CSV format > Click “Save Search Parameters” to save the search created.
