# Supplementary material for: Differential subcellular and extracellular localisations of proteins required for insulin-like growth factor- and extracellular matrix-induced signalling events in breast cancer progression
Source: BMC Cancer. 2014 Aug 29;14:627. doi: 10.1186/1471-2407-14-627 (PMC4158058; doi:10.1186/1471-2407-14-627)
Supplement: Supplementary file 9 — Additional file 9: Semiquantitative evaluation of immunohistochemical immunoreactivity and consolidation of TMA scoring. (DOCX 18 KB) [file 12885_2013_4813_MOESM9_ESM.docx]

**Manuscript title:** Differential subcellular and extracellular localisations of proteins required for insulin-like growth factor- and extracellular matrix-induced signalling events in breast cancer progression.

**Journal name:** BMC Cancer

**Additional file 9:** Semiquantitative evaluation of immunohistochemical immunoreactivity and consolidation of TMA scoring.

A biomarker scoring form was displayed alongside each TMA core for completion. A standard biomarker scoring form was used for all protein targets investigated. Modified biomarker scoring forms were used for ER, PR and HER2 (scoring forms not shown). Specific questions were compulsory. Completion of the scoring forms for each TMA core was required prior to moving on to the next TMA core. Briefly, the TMA scorers were asked to record: their initials; the type of tumour present (provided as a choice between normal breast tissue, DCIS, primary tumour, metastatic tumour tissue from the LN and missing tissue); the amount of tumour present (as a percentage); the quality of the IHC staining (provided as a choice between yes or no); the percentage of cells with cytoplasmic staining (as a percentage); the intensity of the cytoplasmic staining (provided as a choice between 0, 1, 2 or 3); the percentage of cells with nuclear staining (as a percentage); the intensity of the nuclear staining (provided as a choice between 0, 1, 2 or 3); the percentage of cells with membrane staining (as a percentage); the intensity of the membrane staining (provided as a choice between 0, 1, 2 or 3); the percentage of stromal staining (as a percentage); and the intensity of the stromal staining (provided as a choice between 0, 1, 2 or 3).

IHC TMA scoring data was evaluated semiquantitatively using five scoring methods. Details on these scoring methods are listed below.

***Presence:*** Data were expressed as the proportion of the total number of samples in which protein immunoreactivity was present within the normal breast tissue, DCIS, primary tumour or LN metastatic tumour tissue.

***Intensity:*** Data were expressed as a score between 0 and 3, corresponding to the presence of negative, weak, intermediate, or strong staining, respectively, within the normal breast tissue, DCIS, primary tumour or LN metastatic tumour tissue.

***Percentage:*** Data were expressed as the proportion of cells or stroma staining positively within the normal breast tissue, DCIS, primary tumour or LN metastatic tumour tissue.

***Percentage class:*** Data were expressed as scores between 1 and 6 (1 = 0-4%; 2 = 5-19%; 3 = 20-39%; 4 = 40-59%; 5 = 60-79%, and; 6 = 80-100%), corresponding to the proportion of cells staining positively within the normal breast tissue, DCIS, primary tumour or LN metastatic tumour tissue.

***Quickscore (Q score):*** Data were expressed as scores between 0 and 18 using a multiplicative *Q score* system (Detre *et al.* 1995); this involves determining the staining intensity in addition to the proportion of cellular staining. The multiplicative method allows for a “no staining” category.

The exported .csv files from Distiller were converted to Microsoft Excel^™^ spreadsheets for each search performed. Each of the spreadsheet rows corresponded to a TMA core (with a specific tissue type). The variables recorded in the scoring form and the clinico-pathological data was represented as separate columns in the spreadsheet. Duplicate rows represented TMA cores originating from the same tissue sample and placed in duplicate TMAs. If there were multiple TMA scorers then duplication rows would be present for each TMA scorer. If a specific tissue sample was placed into more than one set of duplicate TMAs than duplicate rows would be present for each set of duplicate TMAs. These duplicate rows were consolidated into one row (if possible); the method of consolidation for each variable is listed below. For statistical data analysis purposes, the consolidated rows (each with a specific tissue type) were split up into four rows, each representing the results for a specific cellular localisation.

***Initials:*** If there was only one TMA scorer, the initials of that scorer was recorded in the consolidated row. If there was more than one person who scored the TMA slide for IHC immunoreactivity for a particular protein than the initials of all of the TMA scorers were recorded in the consolidated row.

***The type of tumour present:*** If the type of tissue was the same across the duplicate rows then that type of tissue was recorded in the consolidated row. If there was more than one type of tissue then a consolidated row was required for each tissue type represented.

***The amount of tumour present:*** A mean percentage was calculated based on the proportion of the tissue in the TMA core which contained primary or LN metastatic tumour tissue.

***The quality of the immunohistochemical immunoreactivity:*** TMA cores with unsatisfactory staining were discarded, while TMA cores with satisfactory immunoreactivity were consolidated (by the methods mentioned in this section).

***The percentage of cells with nuclear, cytoplasmic and membrane immunoreactivity:*** A mean percentage was calculated based on the proportion of cells, within the normal breast tissue, DCIS, primary tumour or LN metastatic tumour tissue which contained nuclear, cytoplasmic and/or membrane immunoreactivity.

***The percentage of stromal immunoreactivity:*** A mean percentage was calculated based on the proportion of the stromal cells and ECM (which will be referred to as the ‘stroma’), surrounding the normal breast tissue, DCIS, primary tumour or LN metastatic tumour tissue which contained immunoreactivity.

***The intensity of the nuclear, cytoplasmic, membrane and stromal immunoreactivity:*** The highest intensity score was recorded in the consolidated row.
